# Awareness of the effect of fluctuations in female sex hormone levels on periodontal health among obstetricians

**DOI:** 10.15171/japid.2018.006

**Published:** 2018-06-20

**Authors:** Fahimeh Rashidi Maybodi, Mehrdad Jalali Pandary, Zohreh Rahaei, Shabnam Nikniaz

**Affiliations:** ^1^Department of Periodontics, Shahid Sadoughi University of Medical Sciences, Yazd, Iran; ^2^Private Practice, Yazd, Iran; ^3^Department of Health Education, School of Public Health, Shahid Sadoughi University of Medical Sciences, Yazd, Iran

**Keywords:** Awareness, obstetricians, periodontal health, sex hormones

## Abstract

**Background:**

Changes in women’s hormonal levels in different periods of their lives, such as puberty, menses, pregnancy, menopause and oral contraceptive use, affect periodontal health. Periodontal status has been associated with adverse pregnancy outcomes. Subsequently, it is important for obstetricians to be aware of the effects of hormones on women's oral health. The current study assessed obstetricians’ awareness about the effect of female sex hormones on periodontium in Yazd in 2016.

**Methods:**

A cross-sectional study was conducted using a self-administered, structured questionnaire. Prior to the study, a questionnaire was pre-tested and validated (ICC=0.89). The questionnaire was randomly distributed among 40 obstetricians in Yazd. Data were analyzed with chi-squared test, t-test and ANOVA and Spearman's correlation coefficient, using SPSS 18.

**Results:**

There were no significant relationships between awareness level and the age (P=-0.252), years of practice and experience (P=-0.030) or practicing in public medical centers (P=0.121).

**Conclusion:**

As women have special periodontal health care considerations due to fluctuations in the levels of their sex hormones, this study suggests that promotion of oral health awareness among obstetricians is necessary to improve women's overall health, especially during pregnancy.

## Introduction

Gingivitis and periodontitis are multifactorial diseases predominantly caused by several groups of anaerobic and low-aerobic gram-negative bacteria colonizing periodontal tissues. These bacteria increase the production of proinflammatory mediators such as prostaglandins and cytokines.^1,2^ Several systemic factors can increase the incidence and severity of gingivitis and periodontitis.^3^ Among these factors, changes in sex hormone levels are likely to affect the course of periodontal disease as they change the host microbial flora.^4-7^ For example, bacteria such as *Prevotella intermedia* and *Capnocytophaga* species are more common during puberty.^8,9^ Clinically, hyperplastic reactions might occur in areas of the gingiva where the products of these bacteria accumulate. Female hormones have also special receptors in gingival tissues.^10^ Thus women are at increased risk for oral diseases during puberty, menstruation, pregnancy and menopause as well when they are taking contraceptives.^11^ Some of the usual changes during these different periods of life, which might lead to increased risk of periodontal inflammation, are described below.

In general, estrogen uptake can contribute to epithelial keratinization, new blood vessel formation (angiogenesis), gingival fibroblast proliferation and maturation of gingival connective tissue.^4^ Progesterone also has a direct effect on prostaglandin production and is likely to play an important role in bone resorption rate.^12,13^ In addition to changes during puberty, gingival tissues are erythematous during the menstrual cycle and somehow anesthetic before the cycle begins. As a result, an increase in the tendency for bleeding is observed during menstrual cycles, occasionally accompanied by a slight increase in the mobility of teeth.^14,15^ During pregnancy, the levels of estrogen and progesterone at the end of the third trimester are 10 to 30 times of the amount observed during menstrual cycle, respectively.^16^ Occasionally, local lesions such as pregnancy tumors or granulomas can be seen during pregnancy.^17^ During pregnancy, gingivitis also occurs in up to 36‒100% of pregnant women,^18^ and clinical parameters such as increased bleeding on probing and pocket depth are prevalent during this period with no relationship with the amount of dental plaque.^19^ The need for periodontal therapeutic interventions, according to CPITN (Community Periodontal Index for Treatment Needs) measurements, also increases during pregnancy, indicating the importance of periodontal care during this period.^20^ Changes similar to those mentioned above during pregnancy are seen in women taking oral contraceptives (OCPs).^14^ Women may also experience changes in oral mucosa during menopause, when their hormonal levels decrease, which might cause burning sensation, taste changes, xerostomia or gingivostomatitis.^7,15,18,19^

Considering all these occurrences during puberty, pregnancy, oral contraceptive use and menopause and due to the key role of obstetricians in assessing women over different periods of their lives, the awareness of this group of healthcare providers about the effects of hormonal fluctuations on women's periodontal health is very important. On the other hand, it should be noted that there is an undeniable relationship between general health and oral health according to many studies; in this context, periodontal disease in a patient might increase the odds of diabetes, arthrosclerosis, stroke and myocardial infarction, and in particular, the chances of premature birth or low birth weight.^21-25^

Periodontitis is known as a very common but also curable disease.^26^ Therefore, obstetricians and gynecologists as one of the healthcare providers of women can play an important role in the prevention and treatment of this disease by referring their patients to dentists or by encouraging them to follow oral health instructions.^27^ Unfortunately, few studies have been conducted on the knowledge of gynecologists in this field and to the extent that the authors are aware; there has been no research on their attitude and knowledge about the effect of hormonal changes on oral tissues in our country (Iran). Hence, the aim of the present study was to investigate the awareness of obstetricians in Yazd about the effects of changes in female sex hormones on periodontal health.

## Methods

The protocol of this descriptive cross-sectional study was approved by the Ethics Committee of Shahid Sadoughi University of Medical Sciences under the code IR.SSU.REC.1395.61.

In the present study, 40 gynecologists in Yazd, Iran, were selected through census method. Then, their knowledge about the effects of changes in women's sex hormone levels on periodontal health in different life periods (menstruation, pregnancy and menopause) was measured using a researcher-made questionnaire. Gender, occupational experience, number of years elapsed since graduation of specialists and employment in the private or public sector were also questioned. Finally, the participants were also asked about the main sources of their knowledge in this field.

Data were analyzed with SPSS 18, using t-test, Spearman's correlation coefficient and one-way ANOVA. Statistical significance was set at P<0.05.

The quantitative impact method was used to determine the formal validity of the questionnaire. Scores 1, 2, 3, 4 and 5 were considered for very poor, poor, moderate, strong and very strong options, respectively. Then, the score of each question's impact was calculated using the following formula and the score above 1.5 was considered acceptable:

Impact score=Frequency (%) * Importance

### Questionnaire design

A questionnaire with 12 questions was designed. Before conducting the study, validity and reliability of the questionnaire were evaluated using experts' opinions and implementation of a pilot project on 15 specialists and approved after performing a re-test. The reliability analysis of the questionnaire showed a Cronbach's full-scale alpha value of 89%, with good internal consistency, a split-half reliability and validity.

## Results

The mean age of the participants was 45.77 years (32-73±4.35); the mean of their work experience was 13.83 years (1‒37); and the mean score of their awareness was 13.45. There was no significant relationship between awareness and location of employment (private or public centers) (P=0.121), age (P=-0.252) and work experience (P=-0.030), as follows:

According to Table 1, there was no statistically significant relationship between employment in the private or public sectors and the awareness level of participants.

**Table 1:** Frequency distributions of answers to research questions

**Questions**	**Answers**		
	**Yes** **Number (percentage)**	**No** **Number (percentage)**	**I don’t know** **Number (percentage)**
**Do changes in female sex hormones affect women's gingival status?**	36 (90%)	2 (5%)	2 (5%)
**Does progesterone play a role in increasing the production of inflammatory prostaglandins?**	24 (60%)	8 (20%)	8 (20%)
**Can contraceptive pills, as a long-term prevention method, have a negative effect on the gingiva?**	7 (17.5%)	14 (45%)	19 (47.5%)
**Can the pregnancy itself lead to periodontitis even if the pregnant woman has good oral hygiene?**	22 (55%)	15 (37.5%)	3 (7.5%)

According to Table 1, 90% of the participants believed that changes in female sex hormones affected gingival condition of women. 60% of them believed that progesterone has a role in increasing the production of inflammatory prostaglandins. 17.5% of them were aware of the negative impact of taking oral contraceptives as a long-term preventive method, while 45% of the participants did not agree to this issue. 55% of the participants believed that pregnancy can cause periodontitis even if a pregnant mother has good oral hygiene.

Frequency distribution of answers to some research questions are shown in Table 2; 70% of the participants reported thinning of oral mucosa, 75% of them reported gingival loss and 70% of them reported xerostomia as oral symptoms of the postmenopausal period. Frequency distributions of other answers are shown in Table 3.

**Table 2:** Frequency distributions of answers to research questions

**Questions**	**Answers**	**Number (percent)**
**Which of the following items increases the incidence of gingivitis in women?**	Puberty*	4 (10%)
	Menstruation*	4 (10%)
	Pregnancy*	36 (90%)
	Lactation	8 (20%)
	Menopause*	13 (32.5%)
	None of them	0 (0%)
	I don’t know	4 (10%)
**During which of the following periods hormonal changes can increase the mobility of teeth?**	Puberty	2 (5%)
	Menstruation	3 (7.5%)
	Pregnancy*	22 (55%)
	Lactation	17 (42.5%)
	Menopause	14 (35%)
	None of them	0 (0%)
	I don’t know	6 (15%)
**What is the effect of estrogen reduction on oral tissues?**	Reduces collagen formation in the oral connective tissue*	17 (42.5%)
	Increases vascular proliferation in the gingival tissue	12 (30%)
	Increases glycogen in the gingival tissue	7 (17.5%)
	Reduces keratinization in the gingival tissue*	9 (22.5%)
**Which of the following is related to premenstrual syndrome?**	Nausea*	16 (40%)
	Exaggerated response to pain*	15 (37.5%)
	Increased tendency to sugar consumption*	24 (60%)
	Reduced salivary flow	7 (17.5%)
	Less tolerance during dental treatment*	12 (30%)
	None of them	0 (0%)
	I don’t know	6 (15%)
**In which period of pregnancy the chances of gingival inflammation are high?**	First trimester	8 (20%)
	Second trimester	8 (20%)
	Third trimester*	13 (32.5%)
	No difference	8 (20%)
	I don’t know	3 (7.5%)
**Gingival inflammation during pregnancy increases the chance of which of the following?**	Fetal death	4 (10%)
	Preeclampsia*	12 (30%)
	Premature birth*	33 (82.5%)
	Developmental deficiency	0 (0%)
	Low birth weight*	30 (75%)
	None of them	1 (2.5%)
	I don’t know	3 (7.5%)
**In the presence of gingival inflammation during pregnancy, which of the following can be prescribed if necessary?**	Using mouthwash*	32 (80%)
	Scaling and root planing*	15 (37.5%)
	Amoxicillin*	25 (62.5%)
	Metronidazole	14 (35%)
	I don’t know	3 (7.5%)

**Table 3:** Absolute and relative frequency distributions of responses to the question "Which one is prevalent during menopause?"

**Answer**	**Number (percent)**
Thinning of the oral mucosa*	28 (70%)
Mouth burning*	15 (37.5%)
Gingival recession*	30 (75%)
Xerostomia*	28 (70%)
Changes in taste sensation*	18 (45%)
**Decreased salivary flow**	1 (2.5%)
**None of them**	0 (0%)
**I don’t know**	4 (10%)

*correct answers

## Discussion

Considering the fact that most of the previous studies have not investigated pregnancy, puberty and menopause, the context for comparing the discussion was limited. The results of the present study showed no statistically significant difference between knowledge levels of specialists working in public or private medical centers, which is different from the results reported by Patil et al,^11^ in which there was higher level of knowledge of specialists working in educational centers and public hospitals. Ninety percent of the participants in the present study were aware of the effects of changes in female sex hormones on gingival status. This is different from the results of studies by Cohen^28^ and Patil et al,^11^ which indicated the awareness of 74.7% and 32.98% of participants, respectively, about the effects of women's hormonal changes on gingival health.

This study also showed no statistically significant relationships between the age or the occupational experience of the participants and their level of knowledge, consistent with the results reported by Shah et al,^29^ who emphasized the absence of a statistically significant relationship between age and awareness of participants. On the other side, these results are different from the results reported by Cohen et al,^28^ who reported that the awareness of gynecologists about periodontal diseases increased proportional to their work experience. 82.5% of the participants of the present study considered premature delivery and 75% of them considered low birth weight as the consequences of maternal periodontal disease, which is consistent with the results of studies by Patil et al, Shah et al, Suri, Cohen, Hashim and Laslowski, in which most of the participants considered periodontal disease as a risk factor for adverse outcomes of pregnancy, including low birth weight or premature delivery,^11,28-33^ while in the study by Shenay,^26^ fewer participants considered periodontal disease as a risk factor for inappropriate pregnancy complications despite their proper knowledge about oral manifestations of periodontal diseases. In a study by Bahalla,^34^ the participants only considered vaginal infection as a cause of low birth weight or premature delivery and only reported infectious endocarditis as the main consequence of periodontal disease. In a study by Rahman et al,^35^ less than half of the participants were aware of the relationship between periodontal disease and premature delivery, which is different from the present findings. A study by Nagarakanti^36^ showed low knowledge levels regarding periodontal disease as a risk factor in PLBW, with 10.5% of the respondents considering it to be a risk factor, which is also different from our results. About 30% of participants in our study reported higher prevalence of preeclampsia or pregnancy-related hypertension in the presence of maternal periodontal disease.

Only a small proportion of participants in the present study (17.5%) were aware of the negative effects of contraceptives on the status of periodontium in long-term use (for example, as a usual prevention method), consistent with the study by Patil et al.^11^ This little awareness might be due to the limited number of studies on the relationship between use of oral contraceptives and periodontal health referred in the main textbooks of gynecology courses. 80% of the participants in the present study considered use of oral mouthwashes, 62.5% of them considered amoxicillin prescription and 35% of them considered metronidazole prescription as a safe therapy in the case of gingival inflammation during pregnancy. In Patil et al^11^ study, 61.29% of the participants prescribed mouthwashes, antibiotics and analgesics for their patients if their patients had signs of gingival inflammation. Although these findings can indicate their attention to maintaining of oral health during pregnancy, it should be noted that metronidazole has contraindications in pregnant women and it is associated with an increased risk of spontaneous abortion^37^ and gynecologists need to be well informed about prescribing metronidazole.

According to the present study, the majority of obstetricians and gynecologists were aware of the effects of female hormones on inflammation, including the effect of progesterone on intensification of inflammatory process (60%) and effect of estrogen on reducing the process of repair in gingival tissues, such as collagen changes (42.5%), but few of them (22.5%) were aware of the effect of estrogen on reduction of gingival tissue keratinization.

Most gynecologists (90%) were aware of the effect of hormonal changes on increasing the prevalence of gingivitis during pregnancy but in the study by Nutalapati,^38^ only about half the gynecologists (58.3%) were aware that gingival diseases occur at a higher rate in pregnant women. There was no agreement on the peak time of incidence of gingival changes during pregnancy in our study. About one-third of the participants (32.5%) mentioned that more often there were gingival changes in the third trimester. A significant percentage of participants (32.5%) also believed that changes in menopause are associated with an increased incidence of gingivitis and they were aware of its effect on clinical signs such as gingival recession (75%), oral thinning of the oral cavity and xerostomia (70%) and changes in the taste sensation (45%). However, a small number of participants (10%) indicated the occurrence of gingival changes during puberty and menstruation, indicating a need for more information in this area by gynecologists.

Interestingly, more than half of the participants (55%) thought that pregnancy can still cause periodontitis even if pregnant women have good oral hygiene, while this is a common misconception that periodontitis will not occur if oral hygiene is proper. Regarding the presence of periodontal infection as an independent risk factor for pregnancy adverse outcomes and its curability and preventability, inclusion of periodontal care in women's health programs can prevent unwanted side effects of pregnancy and consequences of other female hormonal changes such as puberty and menopause on periodontal tissues. This study recommends that gynecologists encourage their patients to refer for oral health examinations during their hormonal changes, especially before planning for pregnancy.

## Conclusion

Women have special periodontal healthcare considerations during their sex hormone fluctuations. Our results suggest that an increase in dental health acknowledgment among obstetricians is necessary to improve women’s health and pregnancy outcomes. Gynecologists and obstetricians are recommended to refer pregnant women to dental health centers for routine oral health examinations in addition to other routine check-ups during their pregnancy such as blood tests, anomaly tests, etc.

## Competing interests

The authors declare that they have no competing interests with regards to authorship and/or publication of this work.

## Ethics approval

The Research Ethics Committee of Shahid Sadoughi University of Medical Sciences for this study waived the need for an ethics approval.
